# A Mathematical Model of Myosin Heavy Chain Dynamics in the Disintegration of Golden Threadfin Bream *Nemipterus virgatus* Surimi Gel

**DOI:** 10.3390/gels11050348

**Published:** 2025-05-08

**Authors:** Ryoko Nakamizo, Tatsuya Hayashi, Yuri Kominami, Hideki Ushio

**Affiliations:** 1 Graduate School of Agricultural and Life Sciences, The University of Tokyo, Bunkyo-ku, Tokyo 113-8657, Japanhushio@g.ecc.u-tokyo.ac.jp (H.U.); 2 Fish Protein Laboratory, Suzuhiro Kamaboko Honten Co., Ltd., Odawara 250-0862, Japan; 3 Faculty of Science and Engineering, Yamato University, Suita-shi 564-0082, Japan; hayashi.tatsuya@yamato-u.ac.jp; 4 Research and Development Initiative, Chuo University, Bunkyo-ku, Tokyo 112-8551, Japan

**Keywords:** surimi gel, *Nemipterus virgatus*, gel disintegration, protein gel, mathematical model

## Abstract

Surimi gel, a type of hydrocolloidal food, is formed through the gelation of fish meat proteins. Myosin heavy chain (MHC), a key myofibrillar protein, plays a crucial role in the formation of the gel network via both transglutaminase (TGase)-catalyzed and non-enzymatic polymerization. Gel disintegration in surimi is primarily attributed to the proteolytic degradation of MHC. This study focused on golden threadfin bream *Nemipterus virgatus*, a species characterized by low TGase activity and high protease activity at elevated temperatures. We investigated the competition between non-enzymatic polymerization and proteolytic degradation of MHC and their effects on gel mechanical properties using a mathematical model. A mathematical model based on kinetic reactions accurately reflected the changes in MHC observed through SDS-PAGE analysis during *N. virgatus* gel disintegration. Our results indicate that not only unpolymerized but also polymerized MHC was significantly degraded, which substantially contributed to the reduction in the mechanical properties of the *N. virgatus* surimi. Mathematically understanding the dynamics of MHC in surimi during heating helps promote the utilization of noncommercial fish species for surimi processing by enabling better control over surimi gel properties.

## 1. Introduction

The global demand for fish and fish products has been increasing in parallel with population growth and economic development. To reduce the fishing pressure on commercially important species, the effective utilization of noncommercial or underutilized species is becoming increasingly important. Surimi production represents a practical strategy for utilizing these resources, as surimi can be further processed into a variety of gelled food products, such as fish balls and crab-flavored kamaboko. These surimi-based products are categorized as hydrocolloid gels. During surimi gel production, fish meat is homogenized and its proteins are solubilized under high ionic strength conditions through the addition of salt, followed by gelation mediated by catalytic cross-linking and thermal aggregation of proteins. In surimi gels, myosin heavy chain (MHC), a myofibrillar protein with a molecular weight of approximately 220 kDa, plays a pivotal role in the formation of the gel network [1].

Species-specific differences in the thermal behavior of surimi gel properties complicate surimi processing, particularly due to variations in the temperature at which gelation or disintegration occurs [2,3]. Transglutaminase (TGase) is known as the enzyme that catalyzes the polymerization reaction between MHC molecules through the formation of ε-(γ-Glu)-Lys isopeptide bonds [4,5,6,7]. TGase are widely expressed in the muscle tissues of various fish species, but their activity or temperature dependency is highly species-specific [8]. Conversely, proteases expressed in fish muscle tissues interfere with surimi gelation, and their enzymatic activity and temperature dependency are also highly species-specific [2,3,5,7]. The disintegration of surimi gel due to MHC degradation by proteases can be explained by the following mechanisms: degradation of MHCs proceeds prior to polymerization, known as *modori*, and degradation of polymerized MHCs, referred to as *himodori* [5,9].

To maximize the cross-linking of MHCs catalyzed by TGase and to minimize their proteolytic degradation, a combination of different heating temperatures—known as two-step heating—is commonly employed in the surimi food industry [10]. However, the prediction of surimi gel properties under varying two-step heating conditions remains challenging, as no kinetic model has yet been proposed to explain the dynamics of MHC and the associated mechanical properties during surimi gelation and disintegration. A kinetic model that explains the dynamics of MHC, including the competition among TGase-catalyzed polymerization, non-enzymatic polymerization, and proteolytic degradation, is necessary for a comprehensive understanding of the mechanisms underlying surimi gelation and disintegration. First, it is necessary to develop individual kinetic models that describe *modori* or *himodori* and TGase-catalyzed MHC polymerization, respectively. These models should then be integrated to construct a comprehensive model capable of describing the competitive dynamics among these processes. However, few studies have elucidated the detailed relationship between MHC polymerization/degradation during *himodori* and the mechanical properties of surimi gels, and sufficient empirical data for model construction are still lacking. Therefore, the present study aimed to construct a fundamental model describing the competition between non-enzymatic polymerization and proteolytic degradation of MHC. We focused on golden threadfin bream *Nemipterus virgatus*, which exhibits low TGase activity and high protease activity at elevated temperatures (>50 °C) [3,8,11], and developed a model of MHC dynamics during surimi disintegration caused by *himodori*.

## 2. Results

### 2.1. Effects of the Heating Temperature on the Properties of N. virgatus Surimi Gel

**Figure 1:**
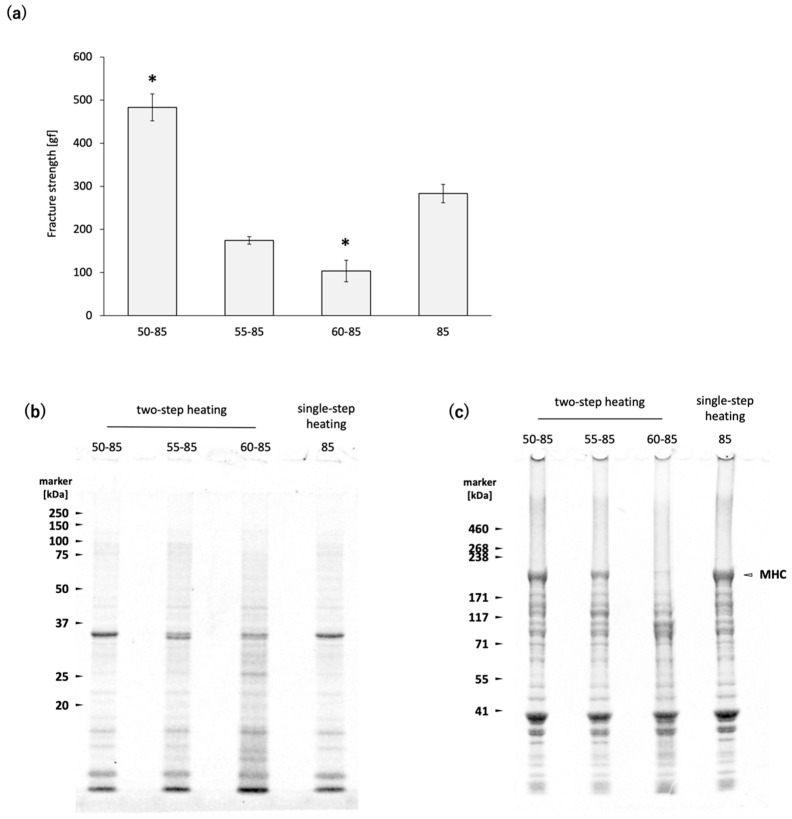
Effects of heating temperature on the properties of N. virgatus surimi gel: (**a**) fracture strength of the surimi gels; (**b**) SDS-PAGE profiles of the WSP; (**c**) SDS-PAGE profiles of the reduced USP. In (**a**), error bars represent the standard error of the mean (SEM) (*n* = 4). * *p* < 0.001 (Dunnett’s test, vs. single-step heating 85).

Figure 1a illustrates the effect of the initial heating temperature on the fracture strength of *N. virgatus* surimi gel. Among the samples, the gel prepared by two-step heating 60–85 exhibited the lowest fracture strength, whereas the gel prepared by two-step heating 50–85 showed the highest.

The separation patterns of the WSP and the reduced USP from the *N. virgatus* surimi gels prepared via two-step heating 50–85, 55–85, and 60–85, as well as single-step heating 85, are shown in Figure 1b,c. In the surimi gel prepared by two-step heating 60–85, the WSP bands appeared smeared, and the band intensity of MHC in the reduced USP was clearly lowest among all samples.

### 2.2. Time Course Analysis of N. virgatus Surimi Gel Disintegration

**Figure 2:**
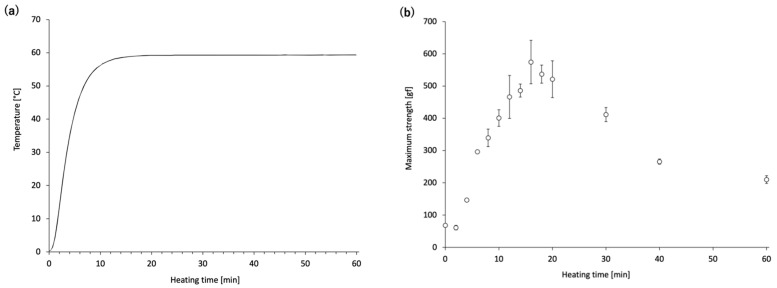
Changes during heating at 60 °C: (**a**) core temperature profile of the surimi sample; (**b**) maximum compression strength as a function of heating duration. Error bars represent the SEM (*n* = 3).

Figure 2a shows the temperature curve near the center of the *N. virgatus* surimi during heating at 60 °C. The core temperature of the 5.5 mL surimi-filled jars reached 60 °C at approximately 16 min. Figure 2b shows the changes in maximum compression strength in relation to the duration of heating at 60 °C. The maximum compression strength rapidly increased with heating duration up to 16 min, after which it gradually declined until 60 min.

### 2.3. Time Course Analysis of Protein Changes in N. virgatus Surimi During 60 °C Heating

**Figure 3:**
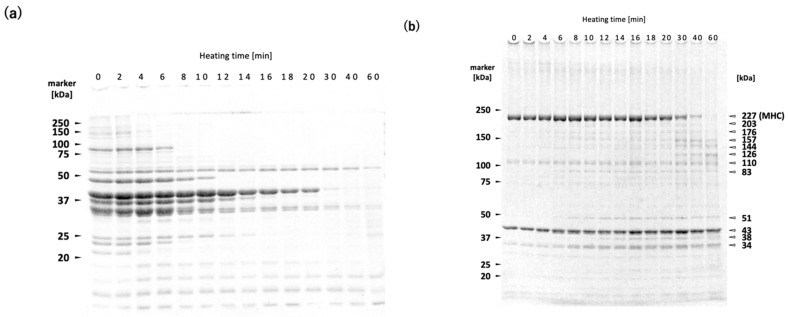

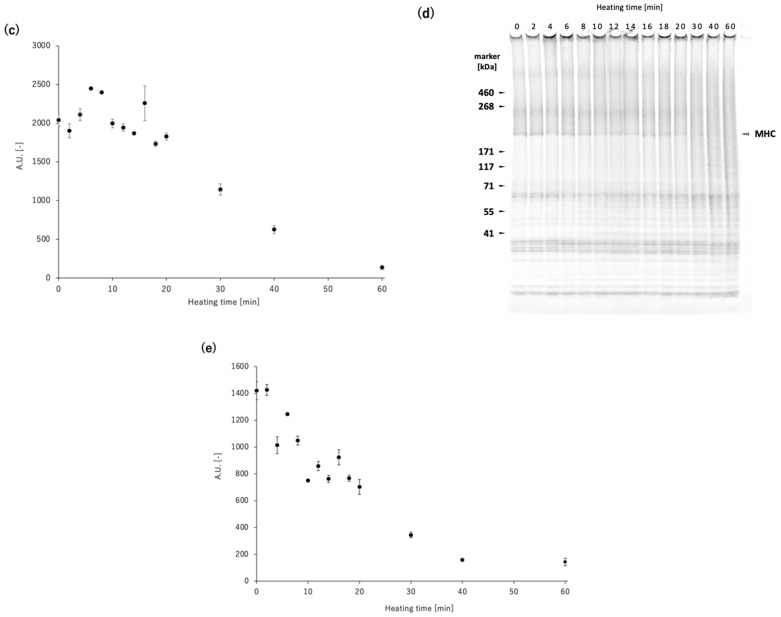
Changes in protein profiles of *N. virgatus* surimi during heating at 60 °C: (**a**) SDS-PAGE profile of the WSP; (**b**) SDS-PAGE profile of the reduced USP; (**c**) MHC band intensities in the reduced USP; (**d**) SDS-PAGE profile of the non-reduced USP; (**e**) MHC band intensities in the non-reduced USP. In (**c**,**e**), error bars represent the standard error of the mean (SEM, *n* = 3), and A.U. denotes arbitrary units (dimensionless).

Figure 3a,b,d show the changes in the WSP, reduced USP, and non-reduced USP profiles of the *N. virgatus* surimi during heating at 60 °C. Figure 3c,e illustrate the quantified MHC band intensities in the reduced and non-reduced USP, respectively. In the WSP, both the number and intensity of protein bands decreased with increasing heating time (Figure 3a). Smeared bands were observed in the WSP from the surimi samples heated for 60 min. In the reduced USP, the intensity of MHC (227 kDa) significantly decreased after 20 min of heating, while bands at 38, 51, 83, 126, 144, 157, 173, and 203 kDa increased (Figure 3b,c). However, after 60 min of heating, the 173 and 203 kDa bands showed a marked decrease. In the non-reduced USP, the intensity of MHC decreased after just 2 min of heating (Figure 3e), and the region above 50 kDa became markedly smeared after 30 min of heating (Figure 3d).

### 2.4. Mathematical Modeling of MHC Non-Enzymatic Polymerization and Proteolytic Degradation

**Figure 4:**
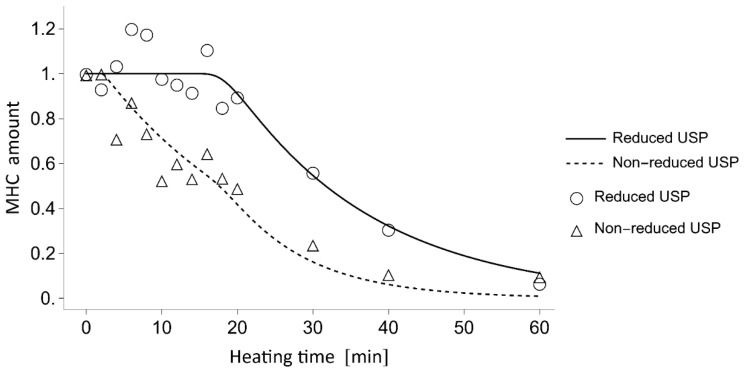
Comparison between the estimated amounts of MHC derived from the mathematical model and the measured MHC band intensities obtained from SDS-PAGE analysis in *N. virgatus* surimi during heating at 60 °C. Solid and dashed lines represent the modeled MHC amounts in the reduced and non-reduced USP, respectively. Circles and triangles indicate the normalized MHC band intensities measured in the reduced and non-reduced USP, respectively.

Figure 4 compares the calculated amounts of MHC with the measured MHC intensities. The MHC band intensities were normalized by setting the initial value to 1 for both the reducing and the non-reducing USP conditions. The calculated changes in MHC levels matched the measured values in both the reduced and non-reduced USP. The decreases in MHC during heating at 60 °C exhibited a delay of approximately 16–18 min in the reduced USP, whereas in the non-reduced USP, the decrease began as early as 2 min.

## 3. Discussion

The present study investigated the competition between the non-enzymatic polymerization and proteolytic degradation of MHC, and their effects on the gelation of golden threadfin bream *N. virgatus* surimi. *N. virgatus* is one of the important fish species used as a raw material for surimi in Asia. Consequently, insights into the *himodori* phenomenon of *N. virgatus* surimi have been accumulated over a long period [3,12,13,14,15,16], and research aimed at improving its gel properties continues to be actively conducted [11,17,18,19,20,21]. First, we confirmed that proteases in *N. virgatus* surimi are activated by heating at 60 °C, which leads to gel disintegration (Figure 1a) [3,13]. The protease responsible for gel disintegration at 60 °C was purified and identified as a sarcoplasmic serine proteinase with a molecular weight of 77 kDa [13]. A sarcoplasmic serine proteinase with a molecular weight of 540 kDa, which has an optimum temperature of 50 °C, was also purified from *N. virgatus* meat [14]. Kinoshita et al. (1990) [3] referred to the protease activated at 60 °C as Sp-60-MIP, and the one activated at 50 °C as Sp-50-MIP. They reported that the *N. virgatus* meat used for the purification of Sp-50-MIP exhibited gel disintegration when heated at 50 °C, but not at 60 °C [14]. In the present study, the *N. virgatus* surimi exhibited gel disintegration when heated at 60 °C, but not at 50 °C (Figure 1a). The results of SDS-PAGE indicate that MHC degradation was clearly promoted at 60 °C, whereas heating at 50 °C did not induce noticeable degradation (Figure 1c). Seasonal variations and freeze-thaw effects are possible factors contributing to this reversal [22,23]; however, further investigation is needed in future studies.

TGase in white croaker *Pennahia argentata*, a species commonly used as surimi ingredients in Japan due to its strong gelation properties, has been reported to show higher peak activity around 30 °C and little to no activity at 50 °C [5,8]. In contrast, TGase in *N. virgatus* meat exhibits relatively lower activity but operates over a broader temperature range, with an optimum around 40 °C [8]. During two-step heating 50, the slower rate of temperature increase compared to single-step heating 85 results in a longer residence time within the temperature range conductive to TGase activity in the *N. virgatus* surimi. Although the *N. virgatus* surimi gel prepared by two-step heating 50 exhibited higher fracture strength than that prepared by single-step heating 85 (Figure 1a), the MHC band intensities remained at comparable levels (Figure 1c). Since TGase-catalyzed MHC polymerization typically results in a decrease in MHC band intensity, as observed by SDS-PAGE, the observed difference in fracture strength between two-step heating 50 and single-step heating 85 cannot be attributed to TGase activity. These results confirmed the negligible activity of TGase during heating at 50–85 °C in the *N. virgatus* surimi used in the present study. The thermal denaturation point of myosin is approximately 40–50 °C in many fish species [24]. During two-step heating 50, the progression from thermal unfolding to aggregation of MHC occurs more gradually compared to single-step heating 85. It has been reported that purified MHC derived from *Cyprinus carpio* forms multimers at 30 °C through association via subfragment-1 [25]. Based on these findings, it is considered that during heating at 50 °C for 60 min, MHC multimerization progresses in the surimi of *N. virgatus*, leading to the formation of MHC clusters, which subsequently bind to each other at 85 °C. This slower polymerization process may contribute to the formation of a finer gel network, thereby enhancing the fracture strength of the surimi gel.

Next, time-course changes in the mechanical properties and MHC monomer levels of the *N. virgatus* surimi during heating at 60 °C were investigated. The maximum strength of the *N. virgatus* surimi reached its highest value after 16 min of heating (Figure 2b), which corresponded to the time when the core temperature of the surimi sample reached 60 °C (Figure 2a). These results suggest that gelation of the surimi progressed until the core temperature reached approximately 60 °C; however, gel disintegration began once the core temperature reached this point.

The WSP decreased with increasing heating time (Figure 3a). The WSP consists primarily of sarcoplasmic proteins, including glycolytic enzymes. It has been shown that WSP of both rainbow trout *Oncorhynchus mykiss* meat and deep-sea bonefish *Pterothrissus gissu* surimi begin to undergo thermal aggregation above approximately 40 °C [26,27]. Although the thermal denaturation point of sarcoplasmic proteins varies depending on fish species and their living environment, it can be estimated to fall within the range of 40–50 °C in *N. virgatus* surimi. Therefore, the decrease in WSP with increasing heating time at 60 °C can be attributed to thermal aggregation rather than proteolytic degradation (Figure 3a). The bands at 34, 38, 51, and 83 kDa observed in the reduced USP are likely sarcoplasmic proteins that became insoluble through thermal aggregation, as indicated by the increase in their band intensities with prolonged heating (Figure 3b).

A decrease in MHC band intensity in *N. virgatus* surimi during heating at 60 °C was observed in both the reduced and non-reduced USP fractions (Figure 3b,d). The decrease observed in the reduced USP indicated proteolytic degradation of MHC, as it was accompanied by an increase in the staining intensities at 126, 144, and 157 kDa (Figure 3b,c). On the other hand, the decrease observed in the non-reduced USP indicates both non-enzymatic polymerization and proteolytic degradation of MHC (Figure 3d,e). The decrease in MHC intensity in the reduced USP began after approximately 20 min of heating (Figure 3b,c), whereas in the non-reduced USP, the decrease was observed as early as 2 min into heating (Figure 3d,e). Taken together, these results suggest that thermal polymerization of MHCs via disulfide bonding occurred prior to their proteolytic degradation in the *N. virgatus* surimi during heating at 60 °C.

To elucidate the competition between non-enzymatic polymerization and proteolytic degradation of MHC in the *N. virgatus* surimi during heating at 60 °C, the experimentally measured values were compared with those calculated using a mathematical model (Figure 4). The model incorporates only non-enzymatic polymerization and proteolytic degradation of MHC, as TGase activity was confirmed to be negligible during heating at 50–60 °C (Figure 1c), as described above. The decrease in MHC levels in the reduced USP during heating at 60 °C, which exhibited a delay of approximately 16 min, was accurately traced by the estimated values (Figure 4). Thus, this delay can be attributed to the temperature-dependent activation of proteases. In both the measured and calculated data, the decrease in MHC levels in the non-reduced USP began as early as 2 min into heating (Figure 4). This result suggests that the 2 min delay before the decrease in MHC level in the non-reduced USP, due to disulfide bonding, can be explained by the time required to reach the myosin denaturation point. A comparison between the MHC dynamics estimated by mathematical modeling and the measured time-course changes in maximum strength indicates that gelation progresses through the non-enzymatic polymerization of MHC prior to the gel disintegration caused by the proteolytic degradation of MHC in *N. virgatus* surimi. Considering the difference in onset time between non-enzymatic polymerization (~2 min) and proteolytic degradation (~16 min), not only unpolymerized but also polymerized MHC, as described in Equation (1), was significantly degraded, which contributed markedly to the reduction in the mechanical properties of the *N. virgatus* surimi.

It has been suggested that proteolytic degradation of the pre-formed gel network (*himodori*) underlies the mechanism of gel disintegration in *N. virgatus* surimi [3,5,13]. The present study quantitatively elucidated these details using a mathematical model. Elucidating the molecular mechanisms underlying surimi gelation and disintegration enables the prediction of gel properties under varying heating temperatures and with the addition of TGase or protease inhibitors. Therefore, our approach may facilitate the utilization of noncommercial fish species for surimi processing by enabling better control over surimi gel properties. However, it is also necessary to construct a mathematical model that can quantitatively describe the relationship between MHC polymerization and degradation, and the mechanical properties of surimi gels.

## 4. Conclusions

The present study proposes a model representing the interplay between the non-enzymatic polymerization and the proteolytic degradation of MHC during gel disintegration in golden threadfin bream *N. virgatus* surimi. The reaction rate of MHC was estimated from a densitometric analysis of SDS-PAGE performed under reducing and non-reducing conditions, and its dynamic behavior was represented by a kinetic model. The changes in mechanical strength during the disintegration of *N. virgatus* surimi can be largely explained by the dynamics of MHC, indicating that the approach presented in this study is effective for quantitatively understanding the gelation process of surimi.

## 5. Materials and Methods

### 5.1. Material

Fresh specimens of *N. virgatus* were purchased at a local market in Tokyo, Japan. The fish were transported to the laboratory on ice. Filleting was performed in a cold room, and the deboned, skinned flesh was stored at −35 °C until further use.

### 5.2. Surimi Preparation

*N. virgatus* surimi was prepared according to the method described by Kominami et al. (2025) [28] with slight modifications. Briefly, frozen flesh was ground with 3% (*w*/*w*) NaCl after thawing overnight at 4 °C. The salt-ground surimi was then packed and sealed into plastic jars of either 20 mL (φ42 × 26 mm) or 5.5 mL (φ29 × 16 mm) capacity. All operations were conducted at 4°C.

The surimi-filled 20 mL jars were subjected to a temperature-controlled water bath heating under the following conditions: 50, 55, and 60 °C for 60 min, followed by heating at 85 °C for 20 min (referred to as two-step heating 50–85, 55–85, and 60–85), or a single-step heating at 85 °C for 20 min (referred to as single-step heating 85). Immediately after heating, the jars were rapidly cooled in ice water. Following overnight storage in ice water, the *N. virgatus* surimi gels were subjected to fracture strength measurements.

The 5.5 mL surimi-filled jars were heated in a 60 °C water bath for varying durations (2, 4, 6, 8, 10, 12, 14, 16, 18, 20, 30, 40, and 60 min), then rapidly cooled and stored overnight in ice water. The temperature near the center of the surimi was monitored during 60 °C heating using a coated thermocouple wire (Type T, outer diameter: 0.9 mm, wire diameter: 0.254 mm), and data were recorded using a multichannel recorder (MCR-4TC, T&D Corporation, Nagano, Japan). Some of the 5.5 mL surimi-filled jars were stored in ice water without heating and used as unheated controls. Both unheated and heated *N. virgatus* surimi samples were subjected to constant-speed compression testing.

### 5.3. Fracture Strength Measurements

The fracture strength of the *N. virgatus* surimi gels was determined using a rheometer, according to the method described by Kominami et al. (2020) [29]. After measurement, the central portion of the sample was excised, flash-frozen in liquid nitrogen, and stored at −80 °C.

### 5.4. Compression Test

A rheometer (FUDOH RHEOMETER, NRM-2010-CW, Rheotech, Tokyo, Japan) was used to perform compression tests. Since non-gelled surimi lacks shape-retaining properties, it must be evaluated while contained in a plastic jar. Therefore, the evaluation method for gelatin described in the Japanese Industrial Standard K6503 was modified accordingly. In this study, to assess gelation at the center of the jar, a plunger with a diameter (φ5 mm) significantly smaller than that of the jar (φ25 mm) was employed. The 5.5 mL plastic jar with its lid removed was secured onto the sample stage. A cylindrical plunger (φ5 mm) was pressed into the surimi-filled plastic jar at a speed of 1 mm/sec to a depth of 10 mm, and the resulting stress transition of the surimi was recorded using a data logger (NR-HA08, KEYENCE, Osaka, Japan). The maximum stress during compression was defined as the maximum compressive stress (gf) for each sample. After measurement, the central portion of the sample was excised, flash-frozen in liquid nitrogen, and stored at −80 °C.

### 5.5. Protein Fractionation and SDS-PAGE

Proteins in the *N. virgatus* surimi gel were fractionated into a water-soluble protein (WSP) fraction and a urea-solubilized protein fraction (USP) according to the method described by Kominami et al. (2025) [28] with slight modifications. The frozen surimi sample was ground to a fine powder within the precooled steel cylinders (TH-SPT, Taitec corporation, Saitama, Japan) of an automatic cryogenic crusher (Taitec Freeze Crusher µT-48, Taitec corporation). The frozen powdered sample was suspended in an ice-cold low ionic strength buffer (20 mM KCl, 100 mM Tris-HCl, pH 7.4) at a concentration of 100 mg/mL, followed by centrifugation at 4000 g for 20 min at 4 °C. The supernatant was collected as the WSP fraction. The remaining pellet was washed twice with the low-ionic strength buffer. Subsequently, 40 mg of the resultant pellet was resuspended in 1 mL of either urea-SDS reducing buffer (0.03% bromophenol blue, 3% SDS, 8 M urea, 2 M thiourea, 75 mM dithiothreitol, 50 mM Tris-HCl, pH 6.8) or urea-SDS non-reducing buffer (0.03% bromophenol blue, 3% SDS, 8 M urea, 2 M thiourea, 50 mM Tris-HCl, pH 6.8). The samples were then solubilized overnight at room temperature by gentle inversion using a benchtop tube rotator. Subsequently, they were centrifuged at 12,000 g for 15 min at 20 °C, and the resulting supernatants were collected as the USP fraction.

The WSP fraction was mixed with 4× Laemmli’s sample buffer (Bio-Rad, Hercules, CA, USA) at a 3:1 ratio. The reduced USP fraction was diluted 10-fold with the urea−SDS reducing buffer. Both samples were heated at 95 °C for 3 min prior to SDS-PAGE. The WSP sample was separated using a 12% poly acrylamide gel, while both reduced and non-reduced USP samples were separated using a gradient 2−15% gel (Multi gel II mini 2/15, Cosmo Bio Co., Ltd., Tokyo, Japan). CBB staining and quantitative analysis were performed as described in a previous study [30]. Briefly, images of the SDS-PAGE bands were acquired using an infrared imaging system (Odyssey Fc Imaging System, LI-COR, Lincoln, NE, USA), configured for an excitation wavelength of 680 nm and an emission wavelength of 700 nm. Densitometric analysis of the acquired images was conducted using ImageJ version 1.51t (US National Institutes of Health, MA, USA).

### 5.6. Data Analysis

Dunnett’s test was conducted using the package “DescTools” in R v3.4.37 to compare the fracture strength of the surimi gels versus those prepared by single-step heating [30,31].

### 5.7. Mathematical Modelling

**Figure 5:**
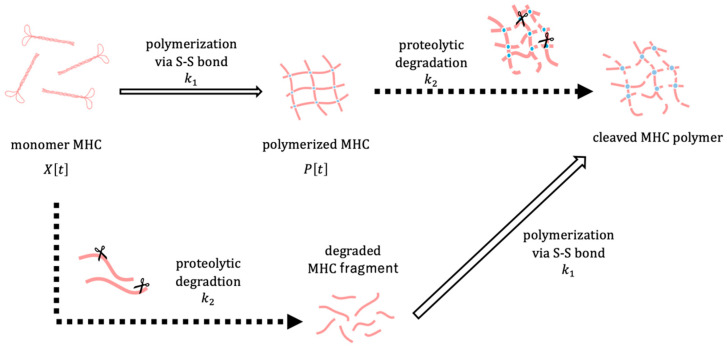
A conceptual illustration of the proposed mathematical model.

The polymerization and degradation of MHC were described by a system of ordinary differential equations. Let X[t] denote the amount of MHC monomer, P[t] the amount of MHC polymer, and T[t] the core temperature of the surimi sample at time t. To focus on relative changes, X[t] and P[t] represent normalized amounts of MHC monomer and polymer, respectively. The conceptual framework of the model is depicted in Figure 5. The amount of MHC in the reduced USP corresponds to the sum of X[t] and *P*[*t*], whereas in the non-reduced USP, it corresponds to X[t] alone. We assume that the polymerization rate of MHC depends on the surimi sample temperature T[t] through a sigmoid function, which reflects the thermal denaturation behaviour of MHC observed in experiments. Considering that MHC degradation is maximized at an optimal temperature, the degradation rate is assumed to be modeled as a Gaussian function dependent on the surimi sample temperature T[t]. The dynamics of MHC polymerization and degradation are described by the following system of differential equations:

(1) $$\begin{array}{c} \frac{dX\left[t\right]}{dt}=-\frac{k_{1}{\left(\frac{T\left[t\right]}{T_{1}}\right)}^{d}}{1+{\left(\frac{T\left[t\right]}{T_{1}}\right)}^{d}}\;X\left[t\right]-k_{2}\exp\left(-\frac{{\left(T\left[t\right]-T_{0}\right)}^{2}}{2\beta^{2}}\right)\;X\left[t\right]\\ \frac{dP\left[t\right]}{dt}=\frac{k_{1}{\left(\frac{T\left[t\right]}{T_{1}}\right)}^{d}}{1+{\left(\frac{T\left[t\right]}{T_{1}}\right)}^{d}}X\left[t\right]-k_{2}\exp\left(-\frac{{\left(T\left[t\right]-T_{0}\right)}^{2}}{2\beta^{2}}\right)\;P\left[t\right]\;\\ \frac{dT\left[t\right]}{dt}=\alpha \left(T_{max}-T\left[t\right]\right)\; \end{array}$$

with the initial values $X\left[0\right]=1$, $P\left[0\right]=0$, and $T\left[0\right]=0$. The model contains the following parameters: $k_{1}$, $k_{2}$, α, β, $T_{0}$, $T_{1}$, $T_{max}$, and d. The parameter $k_{1}$ represents the maximum polymerization rate of MHC. The parameters $T_{1}$ and d are the inflection point and the steepness of the sigmoid function, respectively. The parameter $k_{2}$ denotes the maximum rate of temperature-dependent degradation. The parameter $T_{0}$ is the optimal temperature for MHC degradation and β is the width of a Gaussian function. $T_{max}$ is defined as the highest temperature that the surimi sample can reach, i.e., the temperature of the water bath. The parameter α is the rate at which the heating temperature is increased to approach the target temperature $T_{max}$.

Based on measurements of the thermal denaturation of MHC, the parameters $T_{1}$ and d were estimated as 29.61 and 22.34, respectively, using the Simple Fit app (v4.33) in OriginPro 2024 SR1. The parameters α and $T_{max}$ were set to 0.3 and 60, respectively, from experimental data for the heating temperature. The parameter $T_{0}$ was assigned a value of 60, since the optimal temperature for MHC degradation is assumed to be 60 °C, as supported by our findings (Figure 1) and a previous study [13]. The parameters $k_{1}$, $k_{2}$, and β were determined by grid search to minimize the residual sum of squares between the numerical results and the experimental data. The values of the optimal parameters were found to be $k_{1}=0.044$, $k_{2}=0.053$, and β=0.214. Estimation of the parameters $\left(k_{1},\;k_{2},\;\beta \right)$, numerical solutions to the differential equations, and the data visualization for comparison with the experimental data were obtained using Wolfram Mathematica 13.1.

## Data Availability

The authors confirm that the data supporting the findings of this study are available within the article.
